# Clinical study showing a lower abundance of *Neisseria* in the oral microbiome aligns with low birth weight pregnancy outcomes

**DOI:** 10.1007/s00784-021-04214-x

**Published:** 2021-10-08

**Authors:** Changchang Ye, Meng You, Ping Huang, Zhongyi Xia, Allan Radaic, Jing Tang, Wanhong Wu, Yafei Wu, Yvonne Kapila

**Affiliations:** 1 State Key Laboratory of Oral Diseases, National Clinical Research Center for Oral Diseases, Department of Periodontology, West China Hospital of Stomatology, Sichuan University, Renmin South Road 3rd section 14#, 610041 Chengdu, China; 2 Department of Orofacial Sciences, Division of Periodontology, School of Dentistry, University of California San Francisco, Box 0422, UCSF, San Francisco, CA 94143-0422 USA

**Keywords:** Oral microbiota, Periodontal disease, *Neisseria*, Adverse pregnancy outcomes, Small for gestational age

## Abstract

**Objectives:**

The objective of this study was to examine the association between the oral microbiome and pregnancy outcomes, specifically healthy or preterm low birth weight (PLBW) in individuals with and without periodontal disease (PD).

**Material and methods:**

In this prospective clinical trial, we recruited 186 pregnant women, 17 of whom exhibited PD and delivered PLBW infants (PD-PLBW group). Of the remaining women, 155 presented PD and delivered healthy infants; 18 of these subjects with similar periodontal condition and age matched to the PD-PLBW group, and they became the PD-HD group. From the total group, 11 women exhibited healthy gingiva and had a healthy delivery (HD) and healthy infants (H-HD group), and 3 exhibited healthy gingiva and delivered PLBW infants (H-PLBW group). Periodontal parameters were recorded, and subgingival plaque and serum were collected during 26–28 gestational weeks. For the plaque samples, microbial abundance and diversity were accessed by 16S rRNA sequencing.

**Results:**

Women with PD showed an enrichment in the genus *Porphyromonas*, *Treponema*, and *Filifactor*, whereas women with healthy gingiva showed an enrichment in *Streptococcus*, *Actinomyces*, and *Corynebacterium*, independently of the birth status. Although no significant difference was found in the beta diversity between the 4 groups, women that had PLBW infants presented a significantly lower abundance of the genus *Neisseria*, independently of PD status.

**Conclusion:**

Lower levels of *Neisseria* align with preterm low birth weight in pregnant women, whereas a higher abundance of *Treponema, Porphyromonas*, *Fretibacterium*, and *Filifactor* and a lower abundance of *Streptococcus* may contribute to periodontal disease during pregnancy.

**Clinical relevance:**

The oral commensal *Neisseria* have potential in the prediction of PLBW.

## Introduction

The oral microbiome is a complex microbial community with up to 1,000 total microbial species, comprised of bacteria, fungi, viruses, archaea, and protozoa that live in the oral cavity [1]. Recent data point out the existence of specific microbial patterns that characterize the “healthy oral microbiota,” and these live in homeostasis with the host (eubiosis). However, changes in the oral cavity can alter the oral microbiome balance into a pathogenic state that can promote diseases in the host (dysbiosis) [2]. Current data support that a dysbiosis of the oral microbiome is correlated with the occurrence and progression of oral diseases [3, 4] and systemic diseases, such as cancer, cardiovascular disease [5], diabetes mellitus [6], and adverse pregnancy outcomes (APO) [7–10].

Over the past several decades, significant evidence has emerged that supports an association between periodontal pathogenic bacteria and preterm low birth weight (PLBW) [8, 11, 12]. Specifically, some anaerobic bacteria, such as *Porphyromonas gingivalis* and *Treponema denticola*, have been described as keystone periodontal pathogens and have been used as diagnostic markers of periodontitis, as assessed by traditional culture and PCR methods [3, 13, 14]. Clinical studies indicated that a higher detection frequency of periodontal related microbial species, such as *P. gingivalis*, *Fusobacterium nucleatum*, *Prevotella intermedia*, and *Tannerella forsythia*, in saliva/dental plaque samples were related to PLBW [15]. Moreover, bacterial DNA from *P. gingivalis* and *F. nucleatum* were frequently detected in feto-maternal units, such as placenta and amniotic fluid in mothers with full-term and PLBW neonates [12, 16, 17]. Experimental animal studies showed that oral *P. gingivali*s infection induced preterm birth and low birth weight in pregnant mice. *P. gingivali*s was also observed in the placenta of the infection group by immunohistchemistry [18].

With the recent development of metagenomic sequencing technologies, a growing number of studies have revealed a greater degree of complexity in the oral microbiome than was previously appreciated [19, 20]. Previously, the vaginal microbiome was the most intensely studied area in attempts to find a microbial etiology for adverse pregnancy outcomes. In recent years, literature has expanded into other body sites, such as the placenta, oral cavity, and gut. As a result, evidence from these metagenomic sequencing studies have further characterized several bacterial communities and found that the placenta is most similar to the oral microbiome rather than the vaginal microbiome in terms of taxonomic composition. Moreover, a dysbiosis in the placental microbiome was related to preterm birth. To develop better prediction and intervention approaches for APO, it is critical to understand the oral microbiome changes during pregnancy and their association with APO. This clinical study found that the oral microbiome was relatively stable during pregnancy. However, several genera, such as *Neisseria*, *Porphyromonas*, and *Treponema* were over-represented in the pregnant subjects, while *Streptococcus* and *Veillonella* were more abundant in the non-pregnant women [21]. The contribution of an oral microbiome dysbiosis to APO subjects especially preterm low birth weight has not been well explored.

Therefore, in this study, our objective was to determine the relationships between the oral subgingival microbiome, gingival/periodontal inflammation, and PLBW.

## Material and methods

### Ethical review and informed consent

This study was approved by the Institutional Ethics Committee of West China Hospital of Stomatology, Sichuan University (No WCHSIRB-OT-2016-053) in agreement with the 1964 Helsinki declaration and its later amendments and comparable ethical standards. Subgingival plaque and serum were collected from patients in the Department of Periodontology, West China Hospital of Stomatology, Sichuan University. Written informed consent was obtained from all human subjects who participated in the investigation.

### Study population

In this prospective study, a total of 186 subjects were recruited from the Department of Periodontology, West China Dental Hospital, Sichuan University, from May 2016 to May 2018 including patients and clinical staff. Among these subjects, 17 were diagnosed with periodontal disease (PD) during pregnancy, and they delivered a PLBW infant. These were recruited into the PD-PLBW group. From the total subjects, 155 were diagnosed with PD during pregnancy, and they had a healthy delivery (HD) and delivered a healthy infant (PD-HD-155 group); 18 of these were age and periodontal condition matched with the PD-PLBW group, and they became the PD-HD group (these were selected as the first 18 that were recruited). Among the total subjects, 11 were diagnosed with healthy gingiva during pregnancy, and they had a healthy delivery (HD) and delivered a healthy infant (H-HD group), and 3 subjects were diagnosed with healthy gingiva, and they delivered an PLBW infant (H-PLBW group).

Periodontal examinations were performed during the second trimester of pregnancy. Subgingival plaque and serum samples were collected during the periodontal examination visits. All subjects selected for the study had a minimum of 20 teeth and did not receive any periodontal or antibiotic treatment 3 months before the periodontal examinations. Subjects with any other systemic disease and/or multiple gestations were excluded from the study. Preterm birth (PB) was defined as a gestational age less than 37 weeks. Small for gestational age (SGA) was defined as a birth weight of less than the 10^th^ percentile for gestational age based on Chinese standards [22]. In this study, preterm low birth weight (PLBW) was defined as PB and/or SGA.

### Interview and periodontal examination

All 186 women underwent a full-mouth periodontal examination during 26 to 28 weeks of gestation. Initially, an interview about confounding factors, such as oral hygiene behavior (the frequency of tooth brushing, duration of tooth brushing, utilization of special appliances, utilization of rinse), alcohol consumption, smoking behavior, education level, income level, and health insurance status, was obtained.

Periodontal examinations were then performed by a periodontal specialist with the use of a manual probe (Hu-Friedy, USA). Participants were instructed to abstain from eating and performing oral hygiene procedures for at least 2 h before plaque collection. During the examination, periodontal parameters, including periodontal probing depth (PPD), clinical attachment loss (CAL), and bleeding on probe (BOP), were recorded in six different sites for each tooth, including the mesio-buccal, mid-buccal, disto-buccal, mesio-lingual, mid-lingual, and disto-lingual sites.

### Criteria for periodontal disease (PD) diagnosis in a pregnancy population

Pregnancy seems to have a dramatic effect on the periodontium. Since the 1960s, it has been reported that there is increasing prevalence and severity of gingival inflammation in pregnant women compared to non-pregnant women [23]. Studies have shown that hormonal changes during pregnancy result in greater vascular permeability, gingival edema, crevicular fluid levels, and prostaglandin production, which may lead to increased gingival inflammation [24, 25]. Thus, even though the depth of periodontal pockets seems to increase during pregnancy, the actual PD activity level does not necessarily result in a decrease in the clinical attachment levels [25–27]. Therefore, we used the PD criteria described by Lopez et al. [28] and Vogt et al [25] in this study to operationally select pregnant women who positively and unequivocally exhibited PD—subjects who had gingival redness with 4 or more teeth exhibiting one or more sites of PPD≥4mm and/or BOP at >25% of sites were diagnosed with PD.

Within the PD group, subjects who had CAL>0 were diagnosed with periodontitis (P) and those with CAL=0 were diagnosed as gingivitis (G). Subjects who did not fulfill these criteria were diagnosed as clinically healthy (H).

### Sample collection

Subgingival plaque samples were collected by inserting a sterile #40 paper point for 30 s into the deepest pocket of the Ramfjord teeth [29], which included a right upper molar, an upper incisor, a left upper molar, a right lower molar, a lower incisor, and a left lower molar. When the representative tooth was missing, the next tooth was used, instead. After collection, the samples were stored at −80°C until further use. At 28 weeks of gestational age, peripheral blood was collected and centrifuged at 1500×*g* for 10 min at 4°C to separate the serum. Then, the serum samples were immediately stored at −80°C until further analysis.

### Detection of serum high sensitive-C-reactive protein (hs-CRP)

Serum hs-CRP levels were assessed with the Helica™ C-reactive protein assay ELISA kit (Helica Biosystems, USA) according to the manufacturer’s instructions.

### Microbiome analyses—DNA extraction, PCR amplification, and sequencing

Total DNA was extracted from subgingival plaque samples using the QIAamp® DNA Mini kit (Qiagen, Germany) according to manufacturer’s instructions, and then DNA concentration and purity were determined using a Nano-Drop 2000 UV-vis spectrophotometer (Thermo-Fisher Scientific, USA). DNA samples were then sent to Majorbio Bio-Pharm Technology Co. Ltd. (China) for amplification of the hypervariable V3-V4 regions of the bacterial 16S rRNA gene and sequencing using an Illumina MiSeq (Illumina, USA) platform.

### Microbiome sequencing data analysis

The raw 16S ribosomal rRNA gene reads were demultiplexed and filtered by Trimmomatic trimming tool and merged by FLASH [30]. Operational taxonomic units (OTUs) with 97% similarity cutoff were clustered using the Mothur (http://www.mothur.org). Chimeric sequences were identified and removed to reduce the effects of PCR artifacts, and each OTU representative sequence was classified taxonomically using RDP Classifier (http://rdp.cme.msu.edu), using a confidence threshold of 0.7. Then, alpha diversity (Shannon, Simpson, Chao and Sobs) was obtained using Mothur (http://www.mothur.org). Principal component analysis (PCA) using unweighted UniFrac distance metrics was carried out, and the R package was used to visualize interactions among bacterial communities in different samples.

The 16S rRNA gene sequencing from plaque samples produced more than 2 million raw sequences, and after pre-processing, 2020149 usable sequences with an average of 41,227 ± 8,808 sequences per sample remained in the dataset. The average length of the sequence was 441 bp without the primers, ranging from 388 to 426 bp. After removing the lower credibility OTUs, taxonomic assignment of the sequences resulted in the identification of a total of 1001 OTUs with more than 97% similarity in the subgingival microbiota. Our analysis showed that 99.0% of the oral microbiota sequenced aligned into 12 phyla. Additionally, 95.6% of the oral microbiota were clustered into 99 families and 91.0% aligned with 192 genera.

### Statistics

All statistical analyses were performed using STATVIEW software (Ver. 5.0, SAS Institute, USA). The Friedman’s two-way ANOVA tests were applied to analyze the statistical differences in age, brushing frequency, brushing time, birth weeks, birth weight, mean PPD, mean CAL, percentage of PPD ≥ 5 mm, percentage of BOP-positive sites, and serum hs-CRP concentration between the H-HD, H-PLBW, PD-PLBW, PD-HD, and PD-HD-155 groups and between H, G, and P groups. Chi-square test was performed to compare the dental floss and rinse using rate, education level, income level, health insurance status, and cesarean section rate between the groups. Kruskal–Wallis rank sum test was used to detect comparative taxonomic profiles of different groups at phylum, family, and genus levels, while Spearman correlation coefficients were used to assess the bacterial association with periodontal parameters, hs-CRP, and birth results.

## Results

### Groups had similar demographics except those with PD had lower household incomes

The demographic characteristics of all subjects enrolled in this trial and categorized by their groups (H-HD, H-PLBW, PD-PLBW, PD-HD, and PD-HD-155) are listed in Table 1; H, P, and G groups are listed in Table 2. All recruited subjects reported being between 23 and 36 years old, and none of them reported smoking or alcohol consumption. The household income was significantly lower in subjects with PD, including P and G groups, compared with subjects with healthy periodontal conditions.

**Table 1:** Demographic characteristics of subjects in the H-HD, H-PLBW, PD-PLBW, PD-HD, and PD-HD-155 groups

	H-HD *n*=11	H-PLBW *n*=3	PD-PLBW *n*=17	PD-HD *n*=18	PD-HD-155 *n*=155	*p* value
Age	29.5±3.4	28±5.2	30.6± 5.4	30.1± 4.9	29.9 ± 6.1	NS
Smoking	No	No	No	No	3/155	NS
Alcohol consumption	No	No	No	No	No	NS
Education level (≥12 years)	(8/11) 72.1%	(3/3) 100%	(14/17) 82.3%	(15/18) 83.3%	(123/155) 79.4%	NS
Household income (≥city average)	(9/11) 81.8%	(3/3) 100%	(7/17) 41.1%^*^	(8/18) 44.4%^*^	(79/155) 51.0%^*^	0.022
Medical insurance purchased	(8/11) 72.1%	(3/3) 100%	(11/17) 64.7%	(12/18) 66.7%	(129/155) 83.2%	NS

*means statistically significant (*p*<0.05) difference between sample and H-HD group; NS means statistically not significant difference compared to H-HD group

**Table 2:** Demographic characteristics of subjects in H, P, and G groups

	H (*n*=14)	G (*n*=23)	P (*n*=12)	*p* value
Age	29.5±6.4	30.4±5.3	30.1± 4.	NS
Smoking	No	No	No	NS
Alcohol consumption	No	No	No	NS
Education level (≥12 years)	(11/14) 78.6%	(19/23) 82.6%	(10/12) 83.3%	NS
Household income (≥city average)	(12/14) 85.7%	(10/23) 43.5%^*^	(5/12) 41.7%^*^	0.03
Medical insurance purchased	(11/14) 78.6%	(16/23) 69.6%	(7/12) 58.3%	NS

*means statistically significant (*p*<0.05) difference between sample and H group; NS means statistically not significant difference compared to H group

### Subjects show significant differences in delivery outcomes and clinical periodontitis status

Next, we evaluated the subjects’ periodontal clinical characteristics, serum hs-CRP status, and delivery characteristics, and the results are listed in Tables 3 and 4. Tooth brushing frequency was significantly higher in the subjects with healthy periodontal conditions compared to subjects with periodontitis, whereas dental flossing and use of mouth rinse were equivalent for all groups, which supports the concept that more tooth brushing may have reduced the periodontal parameters.

**Table 3:** Patients oral hygiene behaviors, periodontal parameters, delivery parameters, and inflammation marker levels in the H-HD, H-PLBW, PD-PLBW, PD-HD, and PD-HD-155 groups

	H-HD *n*=11	H-PLBW *n*=3	PD-PLBW *n*=17	PD-HD *n*=18	PD-HD-155 *n*=155	*p* value
Brushing frequency (brushing times per day)	2.3±0.6	3.0±1.0	2.2± 0.4	2.1± 0.6	2.2± 0.6^*^	0.026
Brushing duration (min)	2.3±1.1	2.0±0.0	2.2± 0.7	2.4± 1.3	2.3± 0.9	NS
Dental floss (%)	9.1	0	11.77	11.12	10	NS
Rinse (%)	27.27	33.33	35.29	44.44	30.3	NS
PPD (mm)	1.9±0.3	1.8±0.2	2.6± 0.3*	2.5± 0.4*	2.5± 0.4^*^	0.0001
PPD≥ 5mm (%)	0	0	7.0± 11.8	6.9± 9.2	5.9± 8.4	NS
CAL (mm)	0	0	0.02± 0.02	0.014± 0.02	0.006± 0.01	0.001
BOP (%)	19.3±4.9	23.0±2.1	55.8± 21.6*	53.4± 18.4*	42.9± 18.4^*^	0.0001
Periodontitis (%)	0	0	41.2 (7/17)	44.4 (8/18)	16.1 (25/155)	NS
Delivery time (week)	39.4±1.0	36.5±2.3*	38.2±1.9	39.4±1.2	39.3±0.7	0.005
Birth weight (g)	3475.0±383.5	2436.7±151.8*	2634.7± 160.6*	3341.1± 386.3	3321.8± 378.5	0.001
Cesarean section (%)	63.63	0	58.82	50.0	58.0	NS
hs-CRP (μg/mL)	4.9±2.4	12.9±11.4	5.0±2.1	10.8±26.3	7.4±20.9	NS

*PPD* pocket probing depth, *CAL* clinical attachment loss, *BOP* bleeding on probing; * means statistically significant (*p*<0.05) difference between sample and H-HD group; NS means statistically not significant difference compared to H-HD group

**Table 4:** Patients oral hygiene behaviors, periodontal parameters, delivery parameters, and inflammation marker levels in the H, P, and G groups

	H (*n*=14)	G (*n*=23)	P (*n*=12)	*p* value
Brush frequency (brushings per day)	2.4±0.6	2.2±0.6	2.1± 0.4	0.04
Brush duration (min)	2.3±1.1	2.4±1.2	2.2± 1.0	NS
Dental floss (%)	9.1 (1/14)	8.7 (2/23)	25.0 (3/12)	NS
Rinse (%)	27.27 (3/14)	39.1 (9/23)	50.0 (6/12)	NS
PPD (mm)	1.9±0.3	2.3±0.3	2.7± 0.4*	0.001
PPD≥ 5mm (%)	0	2.0±5.2	13.3± 12.1	0.001
CAL (mm)	0	0	0.03± 0.016*	0.001
BOP (%)	19.3±4.9	45.8±15.4	63.4± 21.9*	0.001
Delivery time (week)	39.4±1.0	38.9±1.4	38.8±1.8	NS
Birth weight (g)	3475.0±383.5	2997.8±492.9*	3102.0± 445.0*	NS
Cesarean section (%)	63.63 (7/14)	47.8 (11/23)	58.3 (7/12)	NS
Preterm birth rate (%)	14.3 (2/14)	8.7 (2/23)	25.0 (3/12)	NS
SGA rate(%)	7.1 (1/14)	43.5 (10/23)*	16.7 (2/12)	0.03
hs-CRP (μg/mL)	4.9±2.4	8.8±23.0	5.5±5.8	NS

*PPD* pocket probing depth, *CAL* clinical attachment loss, *BOP* bleeding on probing; * means statistically significant (*p*<0.05) difference between sample and H group; NS means statistically not significant difference compared to H group

Also, in Table 4, we report the significantly higher incidence of SGA rate in the G group compared to the H group, which led us to test whether oral microbiome dysbiosis had any significant impact on infant delivery.

### Lower abundance of an unclassified *Neisseria* species was found in both PLBW groups compared to HD groups

**Fig. 1.**
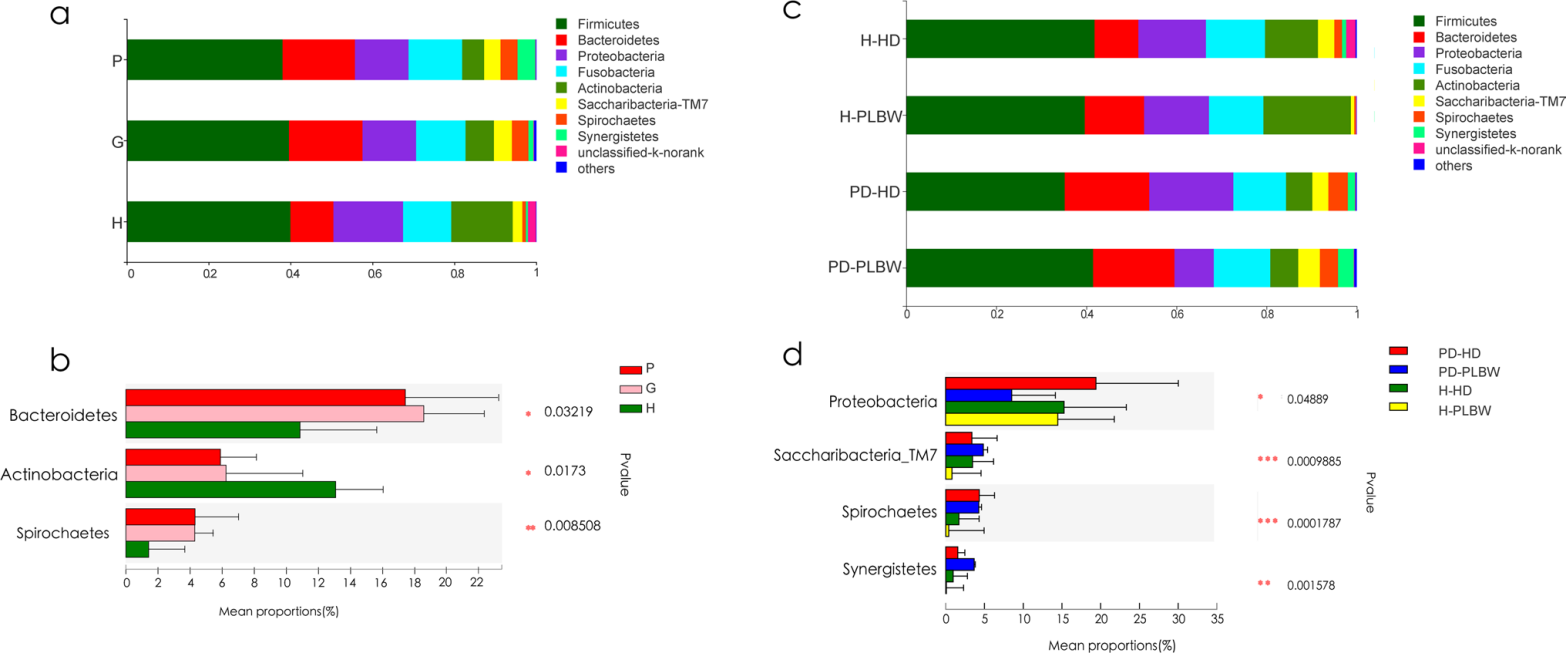
Comparative profiles of phyla found in plaque samples of the H, P, and G groups (**a**) and H-PLBW, H-HD, PD-PLBW, and PD-HD groups (**b**). * represents 0.01 < *p *value ≤ 0.05; ** represents 0.001 < *p *value ≤ 0.01; *** represents *p *value ≤ 0.001

**Fig. 2:**
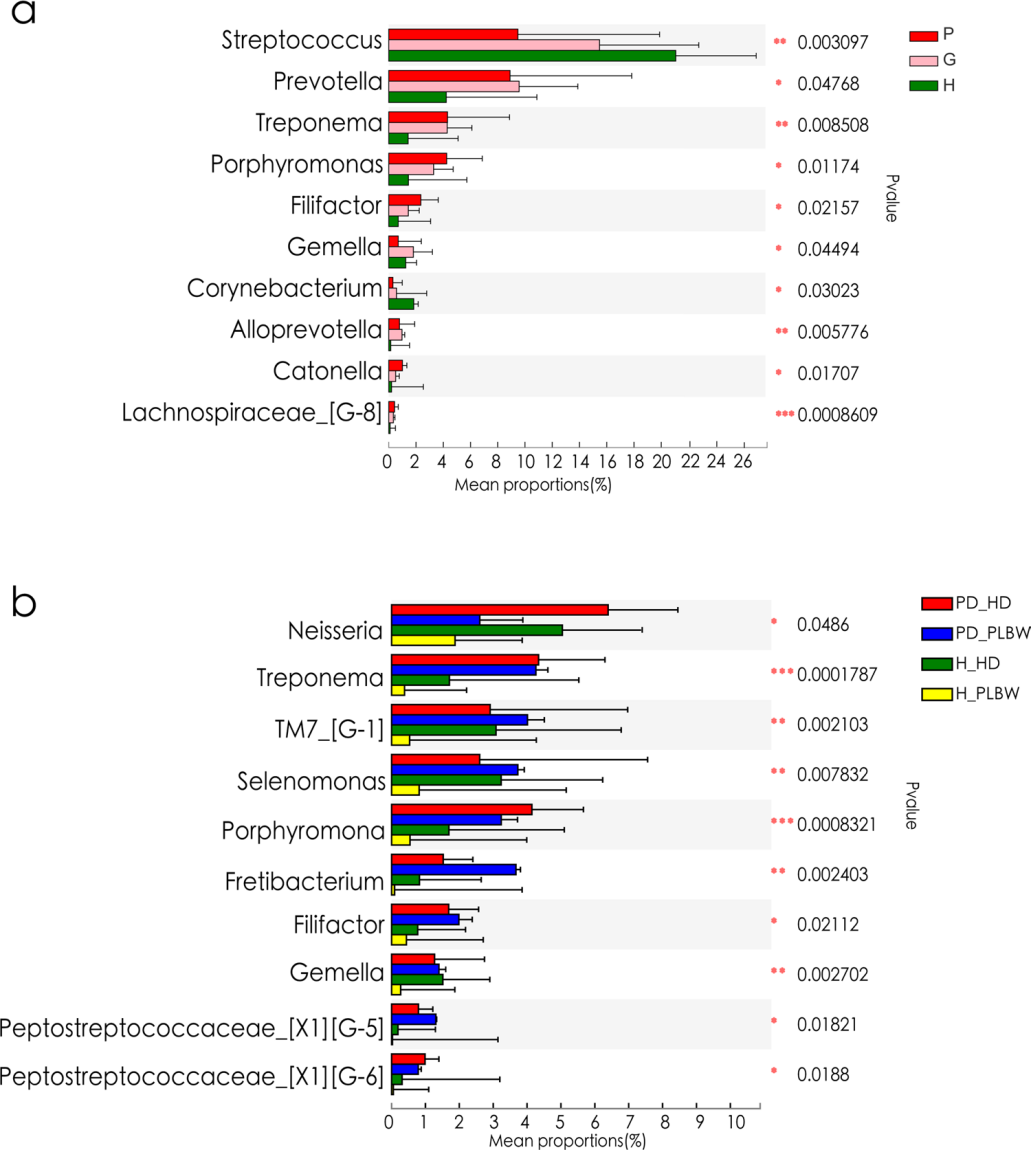
Comparative profiles of genera found in plaque samples of the H, P, and G groups (**a**) and H-PLBW, H-HD, PD-PLBW, and PD-HD groups (**b**). * represents 0.01 < *p *value ≤ 0.05; ** represents 0.001 < *p *value ≤ 0.01; *** represents *p *value ≤ 0.001

**Fig. 3:**
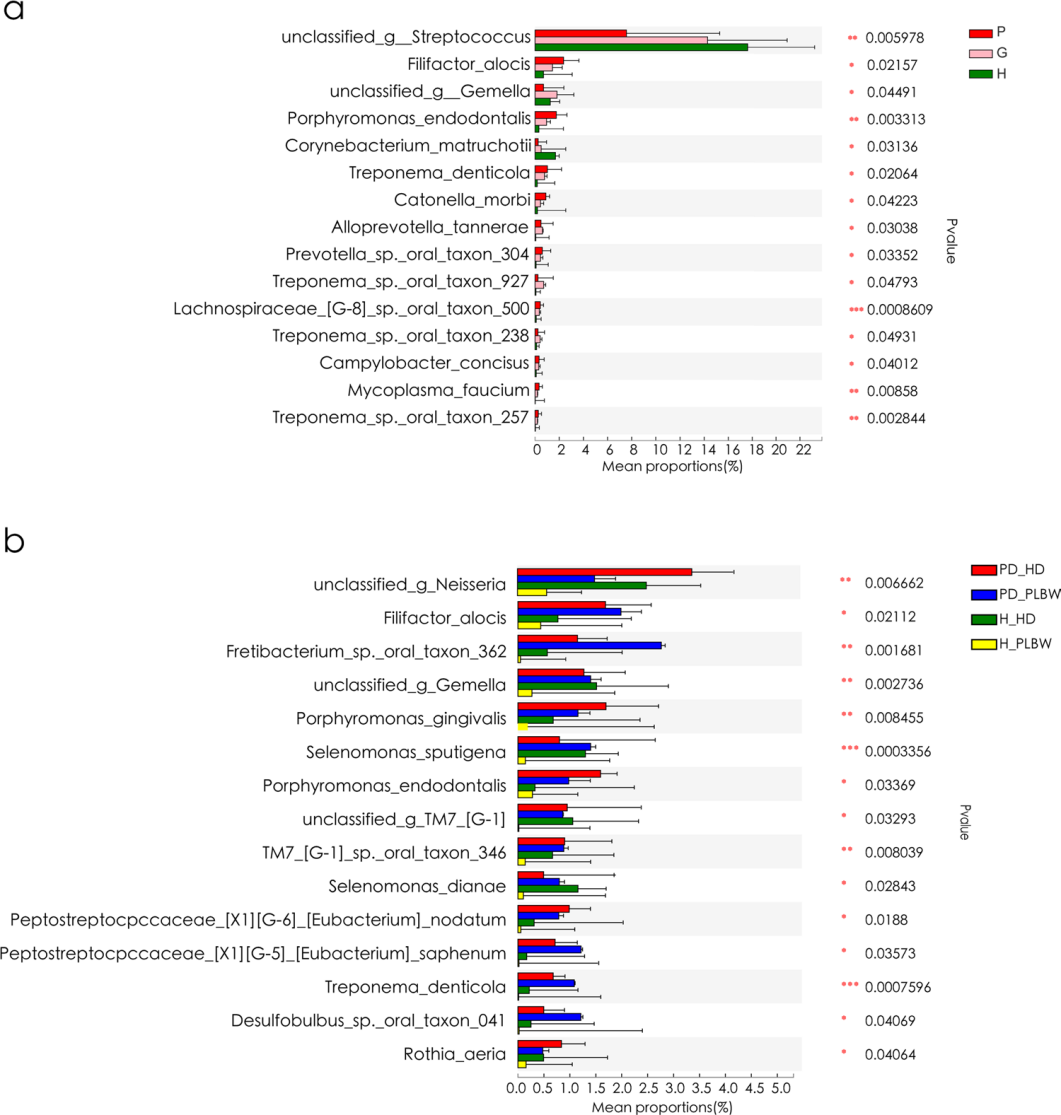
Comparative profiles of species found in plaque samples of the H, P, and G groups (**a**) and H-PLBW, H-HD, PD-PLBW, and PD-HD groups (**b**). * represents 0.01 < *p *value ≤ 0.05; ** represents 0.001 < *p *value ≤ 0.01; *** represents *p *value ≤ 0.001

After determining the periodontal and gestational differences between the groups, we next evaluated the microbial differences between these groups. The results were divided into phyla (Fig. 1), genus (Fig. 2), and species (Fig. 3) level data. At the phylum level (Fig. 1), common oral bacterial phyla, such as *Firmicutes*, *Proteobacteria*, *Fusobacteria*, *Actinobacteria*, *Bacteroidetes*, and *Spirochaetes*, were dominant in all subjects. Nonetheless, *Spirochaetes* and *Bacteroidetes* were significantly higher in both P and G groups compared to both gingivally healthy groups. *Actinobacteria* was lower in both P and G groups compared to the H group (Fig. 1c, d). Interestingly, the PD-PLBW group showed a significant lower *Proteobacteria* content compared to the PD-HD group, suggesting that specific phyla may contribute to PLBW (Fig. 1a, b).

At the genus level (Fig. 2a), *Prevotella*, *Treponema*, *Porphyromonas*, and *Filifactor* were enriched in both P and G groups compared to gingival healthy groups, whereas *Streptococcus* and *Corynebacterium* were enriched in the H group. Interestingly, *Neisseria* were found in lower abundance in the pregnant women that delivered PLBW (PD-PLBW and H-PLBW) compared to the H-HD group (Fig. 2b).

At the species level (Fig. 3a), species such as *Filifactor alocis, Porphyromonas gingivalis*, *Porphyromonas endodontalis*, *Treponema denticola*, *Catonella morbi*, and *Alloprevotella tannerae* were enriched in both P and G groups compared to H group. Unclassified *Streptococcu*s and *Corynebacterium matruchotii* were lower in in both P and G groups compared to the H group.

Interestingly, an unclassified *Neisseria* was found in lower levels in both PLBW groups compared to the healthy delivery groups, possibly driving the lower *Neisseria* genera level in Fig. 3b.

### Despite similar beta diversity, the H-PLBW group appeared more diverse than the PD-HD group

**Fig. 4:**
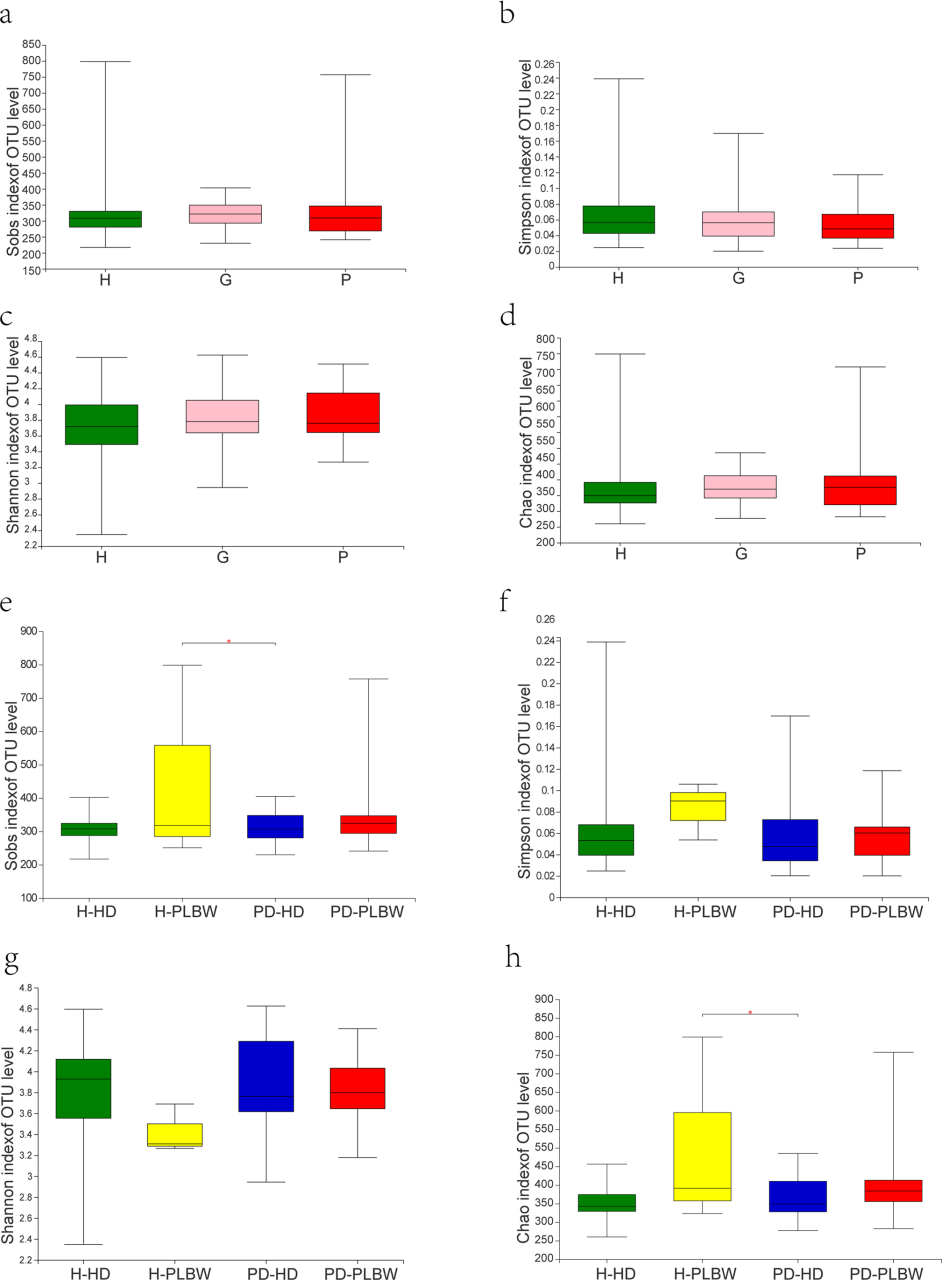
Alpha diversity: the Sobs (**a**), Shannon (**b**), Simpson (**c**), and Chao (**d**) indexes of OTU levels of the H, P, and G groups (**a**, **b**, **c**, **d**) and H-PLBW, H-HD, PD-PLBW, PD-HD groups (**e**, **f**, **g**, **h**). * represents *p value* ≤ 0.05

We next evaluated the alpha diversity of the samples, and the results can be found in Fig. 4. There was no significant difference in the Chao, Sobs, Shannon, and Simpson diversity indices among H, G, and P groups.

Also, no statistical differences were found in the Shannon and Simpson indices among the groups, indicating similar diversities among the H-HD, H-PLBW, PD-PLBW, and PD-HD groups. On the other hand, the Chao and Sobs indices demonstrated a slight but significant increase in diversity for PD-HD group compared to the H-PBLW group. This result may be due to the previously mentioned expansion of the *Actinobacteria* phyla for this sample compared to the other groups.

**Fig. 5:**
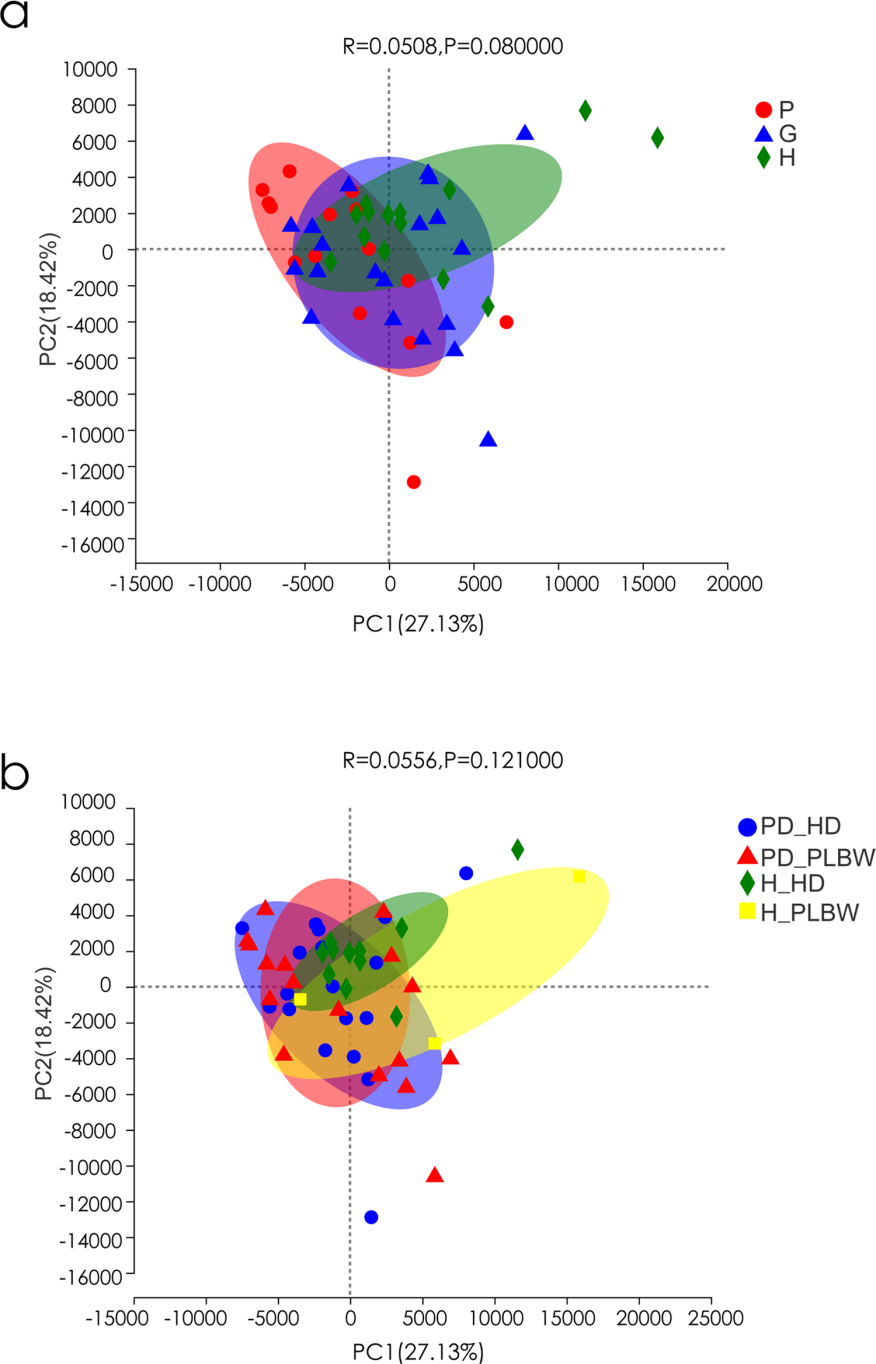
Principal component analysis (PCA) analysis with Bray-Curtis dissimilarity based on genera between the microbiota of the H, P, and G groups (**a**) and H-PLBW, H-HD, PD-PLBW, and PD-HD groups (**b**). Points represent samples in each group

We next evaluated the beta diversity using principal component analysis (PCA) of the genus level data, and the results can be seen in Fig. 5. The analysis showed no obvious separation between the H, G, and P groups and between the H-HD, H-PLBW, PD-PLBW, and PD-HD groups, which corroborated the alpha diversity results, indicating similar microbial community structures for all groups. However, the H-HD group seemed to have a tight arrangement compared to the diseased groups and those with PBLW outcomes (i.e., PD-HD, H-PBLW, and PD-PBLW), which could indicate increased diversity in the diseased groups compared to H-HD group. Among the groups, the H-PLBW group seemed to be the most expanded, which agrees with the significantly higher diversity in the Chao and Sobs indices. Nonetheless, no significant difference was found among the groups.

### *Neisseria* genus is positively associated with birth weight

**Fig. 6:**
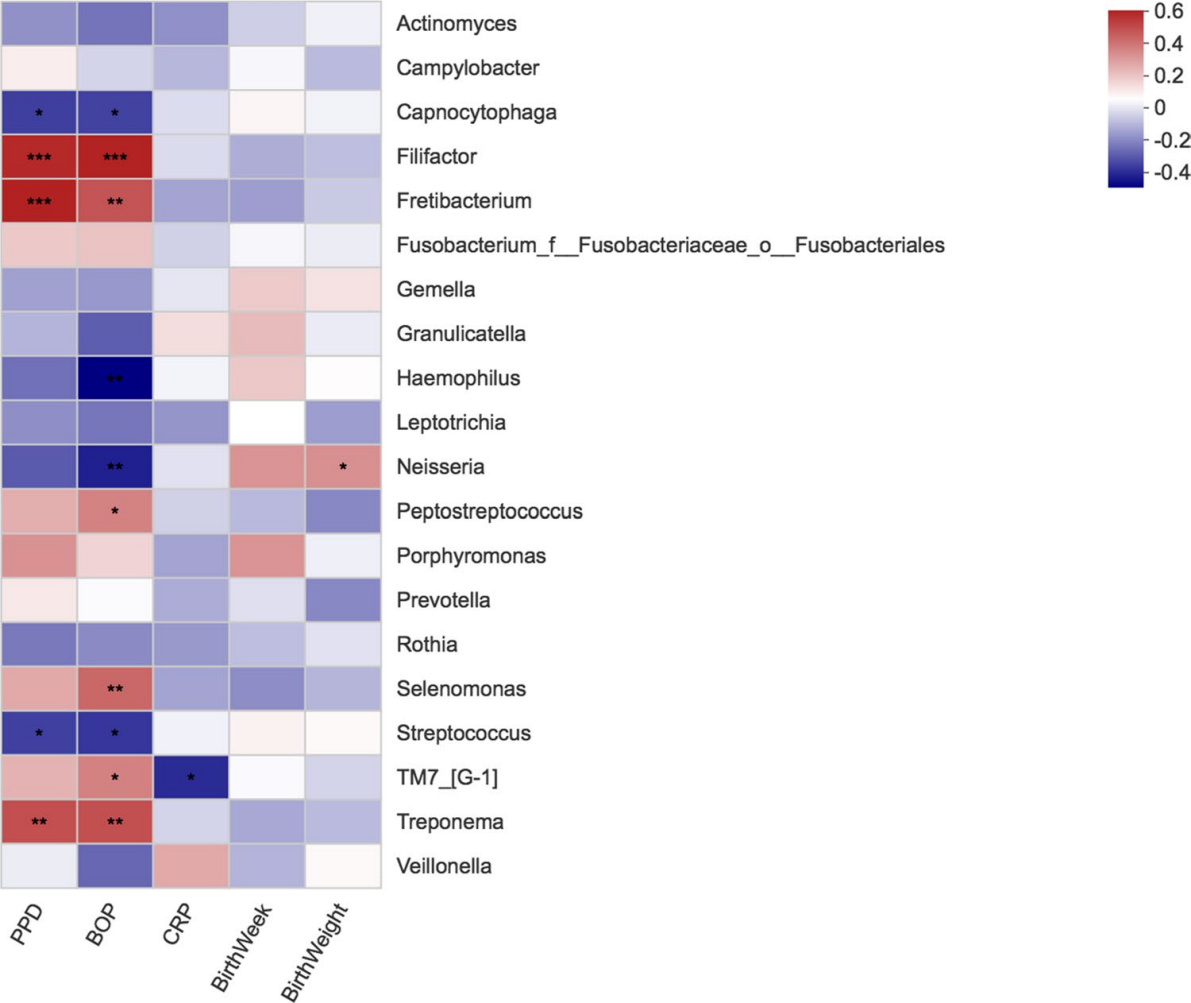
The heat map of Spearman’s correlation coefficient (*R*) between top 20 genus bacteria with periodontal, inflammation, and delivery parameters. Negative correlation, blue; positive correlation, red; * represents 0.01 < *p *value ≤ 0.05; ** represents 0.001 < *p *value ≤ 0.01; *** represents *p *value ≤ 0.001

After determining the alpha and beta diversities, we evaluated the association of the top 20 genera with general inflammation, and periodontal and delivery parameters and the results can be found in Fig. 6. The results indicated that *Capnocytophaga*, *Haemophilus*, and *Streptococcus* genera were negatively correlated with periodontal parameters, such as PPD and BOP, whereas *Filifactor*, *Fretibacterium*, *Peptostreptococcus*, *Selenomonas*, *TM7*, and *Treponema* were positively correlated the same parameters. These data indicate that different microbial groups may be modulating the health/disease status of the periodontal tissues, with *Capnocytophaga*, *Haemophilus*, *Neisseria*, and *Streptococcus* genera possibly protecting the host whereas *Filifactor*, *Fretibacterium*, *Peptostreptococcus*, *Selenomonas*, *TM7*, and *Treponema* genera possibly aggravating/degrading hosts tissues and promoting periodontitis. However, *TM*7 was also negatively correlated with the general inflammation marker hs-CRP.

These data further indicate that *Neisseria* is positively associated with birth weight, while being negatively associated with the periodontal disease parameter BOP. This result suggests a possible protective role for *Neisseria* in both periodontitis and during delivery. This finding is in agreement with the lower abundance of the *Neisseria* genus and of an unclassified *Neisseria* species in the PBLW samples.

## Discussion

Intrauterine infection is the primary cause of APO. Evidence indicates two possible origins of intrauterine infections: ascending infection from the lower genital tract and hematogenous transmission from the oral microbiome. Although current paradigms suggest that most intrauterine infections originate in the lower genital tract and ascend into an otherwise “sterile” intrauterine environment, recent evidence showed that the placental microbiome was composed of nonpathogenic commensal microbiota from the *Firmicutes*, *Tenericutes*, *Proteobacteria*, *Bacteroidetes*, and *Fusobacteria* phyla. This profile is distinct from that of the vagina but similar to that of the oral microbiome [7, 31].

Findings from the last 2 decades supported a long-standing strong association between maternal periodontal disease and increased risk of pregnancy outcomes [32, 33]. There are two possible pathogenic mechanisms that might explain the effect of periodontal inflammation on pregnancy outcomes [34]. First, periodontal pathogens originating in the gingival biofilm directly affect the feto-placental unit subsequent to bacteremia. Second, inflammatory mediators secreted by the subgingival inflammatory site are carried to the feto-placental unit, where they then cause an inflammatory response. So far, evidence indicates that periodontal pathogens, especially *P. gingivalis*, are strongly associated with APO, such as preterm birth and low birth weight. Clinical evidence shows that higher amounts of *P. gingivalis* in subgingival plaque increase the risk of preterm birth, including our previous studies [35, 36]. Animal studies confirm that oral infection with *P. gingivalis* induce preterm birth and low birth weight [18, 37].

An important key point of this study is that we were able to match the age and the periodontal status of 18 subjects, which would assist us in understanding how the oral microbiome might differently contribute to the birth results, without these other confounding variables.

A significant finding of this study is the possible correlation of the *Neisseria* genera and an unclassified *Neisseria* species in patients with gingival/periodontal inflammation and preterm low birth weight. We specifically found the genus to be negatively related with bleeding on probe (BOP) and positively related to birth weight, whereas the Neisseria genus and an unclassified *Neisseria* species were significantly lower in subjects with PLBW delivery. *Neisseria* is one of the most predominant genera among the *Proteobacteria*, which itself is one of the four most abundant phyla colonizing the oral mucosa of humans along with *Firmicutes*, *Bacteroidetes*, and *Actinobacteria* [38].

Several studies have evaluated the protective role of *Neisseria* species in oral diseases. Yamashita et al. [39] and Meuric et al. [40] reported that the predominance of the genus *Neisseria* in oral microbiome indicates healthy periodontal conditions. A study in children between 3 and 18 years of age revealed that *Neisseria* is highly prevalent (97% of the total saliva samples) and *Neisseria flavescens* has been associated with a caries-free status [41]. Zaura et al. [42] identified *Neisseria* as part of the healthy “core microbiome” of the human oral cavity. In contrast, Colombo et al. reported that *Neisseria* species were related to periodontal inflammation and tissue destruction [43].

The *Neisseria* genera have also been studied in relation to systemic diseases. For instance, Said et al. [44] reported a significant lower abundance of *Neisseria* and *Gemella* genera in the salivary microbiota of inflammatory bowel disease patients compared with healthy ones. Interestingly, Peters et al. [45] reported that the commensal genus *Neisseria* and the *Streptococcus pneumoniae* species were associated with lower esophageal cancer risk, whereas Farrell et al. [46] reported that *Neisseria elongata*, along with *Streptococcus mitis*, were significantly less abundant in pancreatic cancer patients compared to healthy patients. In contrast, the *Neisseria* genus was reported to be enriched in HIV+ subjects regardless of sampling site and PD level [47], which might indicate that some species in the genera may be opportunistic pathogens in humans. For instance, *Neisseria gonorrhoeae* is one of the most well studied pathogenic *Neisseria* species. The species can colonize the oropharynx, causing an infection, but without symptoms. Yet, this infection can be transmitted during sexual activities to other sites of the body that are more typically associated with infectious complications, such as pelvic inflammation, epididymitis, and adverse pregnancy outcomes. Interestingly, the nonpathogenic *Neisseria* commensal bacteria can negatively affect *N. gonorrhoeae* colonization by bacterial interspecies competition. Kim et al. reported that nonpathogenic *Neisseria* commensal bacteria, such as *Neisseria elongata* can inhibit *N. gonorrhoeae* by a DNA uptake-dependent manner and pathogen clearance in vivo [48].

However, the influence of *Neisseria* on pregnancy outcomes is still uncertain. Lin et al. reported an increased abundance of *Neisseria*, *Porphyromonas*, and *Treponema* in the supra-gingival plaque samples of pregnant women, whereas *Streptococcus* and *Veillonella* genera were more abundant in the non-pregnant group [21]. Conversely, Paropkari et al. reported that pregnancy was associated with a significant decrease in species belonging to *Neisseria* and *Aggregatibacter* when compared to the non-pregnancy group [49]. Therefore, more studies are needed to explore this apparent discrepancy and to determine the impact of the *Neisseria* genus in pregnancy. Nonetheless, this study addresses the role of the nonpathogenic *Neisseria* species in adverse pregnancy outcomes.

Our data also supports that the intrinsic composition of bacterial taxa changes between pregnant women with or without gingival/periodontal inflammation and between women with healthy delivery and preterm low birth weight delivery. Prospective studies have shown that the level of *P. gingivalis*, *T. denticola*, *P. intermedia*, *T. forsythia*, *Campylobacter rectus*, *A. actinomycetemcomitans*, and *Fretibacterium* sp. HOT360 in the oral microbiome was positively correlated with gingival inflammation during pregnancy [50–52]. In this study, in terms of the composition of the oral microbiota, *Firmicutes*, *Proteobacteria*, *Bacteroidetes*, *Fusobacteria*, and *Actinobacteria* were the five dominant phyla in the mouth. A higher abundance of *Spirochaetes* and *Bacteroidetes* and a lower abundance of *Actinobacteria* were seen in groups with gingival/periodontal inflammation compared with gingival/periodontal healthy groups. Nonetheless, at the genus level, *Treponema*, *Porphyromonas*, *Fretibacterium*, *Filifactor*, and *Peptostreptococcaceae* were associated with gingival/periodontal inflammation. Periodontal therapy, including non-surgical periodontal therapy or probiotics may be warranted before or during pregnancy to reduce periodontal inflammation and correct the maternal oral microbiome dysbiosis.

We found no significant changes in microbiome diversity between the four groups, which is in agreement with the literature. For instance, Yang et al. [53] indicate that the oral microbiome diversity was stable during pregnancy and gingival inflammation and birth outcomes might not be related to overall microbial community structures shifts. Also, we found no significant changes between P and G group, a possible reason might be related to the subjects’ age. All subjects were pregnant women in their 20s to 30s with minimal CAL.

This study has some limitations. One of the limitations of this study was the small sample size, especially in the H-PLBW group. This study was performed at a single institution, which prevented us from having a larger sample size. The oral microbiome during pregnancy appears to be complex, and this fact by itself warrants further large-scale longitudinal studies to understand the role of oral bacteria in PLBW outcomes. Additionally, single-variate statistical analyses were used in this study, which could have limited the overall results of the study.

## Conclusion

In this study, we found a lower abundance of the *Neisseria* genus and of an unclassified *Neisseria* species in women who had preterm low birth weight deliveries. Moreover, our analysis demonstrated that *Neisseria* genera were positively correlated to birth weight in these women. Therefore, correcting oral microbiome dysbiosis, such as improving *Neisseria* genera abundance before or during pregnancy by periodontal therapy, may be useful in preventing PLBW deliveries. Thus, the oral commensal *Neisseria* may have a clinical potential for predicting PLBW.

## References

[1] Lamont RJ, Koo H, Hajishengallis G (2018). The oral microbiota: dynamic communities and host interactions. Nat Rev Microbiol.

[2] Allan Radaic YK (2021) The oralome and its dysbiosis: new insights into oral microbiome-host interaction. Computational Struct Biotechnol 10.1016/j.csbj.2021.02.010 PMC7960681 33777334

[3] Hajishengallis G, Lamont RJ (2012). Beyond the red complex and into more complexity: the polymicrobial synergy and dysbiosis (PSD) model of periodontal disease etiology. Mol Oral Microbiol.

[4] Jiao Y, Hasegawa M, Inohara N (2014). The role of oral pathobionts in dysbiosis during periodontitis development. J Dent Res.

[5] Kholy KE, Genco RJ, Van Dyke TE (2015). Oral infections and cardiovascular disease. Trends Endocrinol Metab.

[6] Ohlrich EJ, Cullinan MP, Leichter JW (2010) Diabetes, periodontitis, and the subgingival microbiota. J Oral Microbiol 2, doi: 10.3402/jom.v2i0.5818 10.3402/jom.v2i0.5818 PMC3084563 21523215

[7] Aagaard K (2014). The placenta harbors a unique microbiome. Sci Transl Med.

[8] Cobb CM (2017). The oral microbiome and adverse pregnancy outcomes. Int J Womens Health.

[9] Figuero E, Han YW, Furuichi Y (2020). Periodontal diseases and adverse pregnancy outcomes: mechanisms. Periodontol.

[10] Bobetsis YA, Graziani F, Gursoy M, Madianos PN (2020). Periodontal disease and adverse pregnancy outcomes. Periodontol.

[11] Han YW, Wang X (2013). Mobile microbiome: oral bacteria in extra-oral infections and inflammation. J Dent Res.

[12] Ye C et al (2020) The periodontopathic bacteria in placenta, saliva and subgingival plaque of threatened preterm labor and preterm low birth weight cases: a longitudinal study in Japanese pregnant women. Clin Oral Investig. 10.1007/s00784-020-03287-4 10.1007/s00784-020-03287-4 32333174

[13] Socransky SS, Haffajee AD, Cugini MA, Smith C, Kent RL (1998). Microbial complexes in subgingival plaque. J Clin Periodontol.

[14] Salminen A (2015). Quantitative PCR analysis of salivary pathogen burden in periodontitis. Front Cell Infect Microbiol.

[15] Swati P (2012). Simultaneous detection of periodontal pathogens in subgingival plaque and placenta of women with hypertension in pregnancy. Arch Gynecol Obstet.

[16] Ercan E (2013). Evaluation of periodontal pathogens in amniotic fluid and the role of periodontal disease in pre-term birth and low birth weight. Acta Odontol Scand.

[17] Wang X (2013). Comparative microbial analysis of paired amniotic fluid and cord blood from pregnancies complicated by preterm birth and early-onset neonatal sepsis. PLoS One.

[18] Ao M (2015). Dental infection of Porphyromonas gingivalis induces preterm birth in mice. PLoS One.

[19] Park OJ (2015). Pyrosequencing analysis of subgingival microbiota in distinct periodontal conditions. J Dent Res.

[20] Hong BY (2015). Microbiome profiles in periodontitis in relation to host and disease characteristics. PLoS One.

[21] Lin W (2018). Ecological shifts of supragingival microbiota in association with pregnancy. Front Cell Infect Microbiol.

[22] Chen Y, Wu L, Zou L, Li G, Zhang W (2017). Update on the birth weight standard and its diagnostic value in small for gestational age (SGA) infants in China. J Matern Fetal Neonatal Med.

[23] Wu M, Chen SW, Jiang SY (2015). Relationship between gingival inflammation and pregnancy. Mediators Inflamm.

[24] Amar S, Chung KM (1994). Influence of hormonal variation on the periodontium in women. Periodontol.

[25] Vogt M, Sallum AW, Cecatti JG, Morais SS (2012). Factors associated with the prevalence of periodontal disease in low-risk pregnant women. Reprod Health.

[26] Moss KL, Beck JD, Offenbacher S (2005). Clinical risk factors associated with incidence and progression of periodontal conditions in pregnant women. J Clin Periodontol.

[27] Taani DQ, Habashneh R, Hammad MM, Batieha A (2003). The periodontal status of pregnant women and its relationship with socio-demographic and clinical variables. J Oral Rehabil.

[28] Lopez NJ, Smith PC, Gutierrez J (2002). Higher risk of preterm birth and low birth weight in women with periodontal disease. J Dent Res.

[29] Ramfjord SP (1959). Indices for prevalence and incidence of periodontal disease. J Periodontol.

[30] Bolger AM, Lohse M, Usadel B (2014). Trimmomatic: a flexible trimmer for Illumina sequence data. Bioinformatics.

[31] Gomez-Arango LF (2017). Contributions of the maternal oral and gut microbiome to placental microbial colonization in overweight and obese pregnant women. Sci Rep.

[32] Ide M, Papapanou PN (2013). Epidemiology of association between maternal periodontal disease and adverse pregnancy outcomes--systematic review. J Clin Periodontol.

[33] Offenbacher S (1996). Periodontal infection as a possible risk factor for preterm low birth weight. J Periodontol.

[34] Offenbacher S (1998). Potential pathogenic mechanisms of periodontitis associated pregnancy complications. Ann Periodontol.

[35] Ryu JI (2010). Health behaviors, periodontal conditions, and periodontal pathogens in spontaneous preterm birth: a case-control study in Korea. J Periodontol.

[36] Ye C (2013). The anti-phospholipid antibody-dependent and independent effects of periodontopathic bacteria on threatened preterm labor and preterm birth. Arch Gynecol Obstet.

[37] Liang S (2018). Periodontal infection with Porphyromonas gingivalis induces preterm birth and lower birth weight in rats. Mol Oral Microbiol.

[38] Contreras M (2010). The bacterial microbiota in the oral mucosa of rural Amerindians. Microbiology (Reading).

[39] Yamashita Y, Takeshita T (2017). The oral microbiome and human health. J Oral Sci.

[40] Meuric V et al*.* (2017) Signature of microbial dysbiosis in periodontitis. Appl Environ Microbiol 83, doi:10.1128/AEM.00462-17 10.1128/AEM.00462-17 PMC5494626 28476771

[41] Crielaard W (2011). Exploring the oral microbiota of children at various developmental stages of their dentition in the relation to their oral health. BMC Med Genomics.

[42] Zaura E, Keijser BJ, Huse SM, Crielaard W (2009). Defining the healthy “core microbiome” of oral microbial communities. BMC Microbiol.

[43] Vieira Colombo AP, Magalhaes CB, Hartenbach FA (2016). Martins do Souto, R. & Maciel da Silva-Boghossian, C. Periodontal-disease-associated biofilm: a reservoir for pathogens of medical importance. Microb Pathog.

[44] Said HS (2014). Dysbiosis of salivary microbiota in inflammatory bowel disease and its association with oral immunological biomarkers. DNA Res.

[45] Peters BA (2017). Oral microbiome composition reflects prospective risk for esophageal cancers. Cancer Res.

[46] Farrell JJ (2012). Variations of oral microbiota are associated with pancreatic diseases including pancreatic cancer. Gut.

[47] Noguera-Julian M (2017). Oral microbiome in HIV-associated periodontitis. Med (Baltimore).

[48] Kim WJ (2019). Commensal Neisseria kill Neisseria gonorrhoeae through a DNA-dependent mechanism. Cell Host Microbe.

[49] Paropkari AD, Leblebicioglu B, Christian LM, Kumar PS (2016). Smoking, pregnancy and the subgingival microbiome. Sci Rep.

[50] Yokoyama M (2008). Relationship between Campylobacter rectus and periodontal status during pregnancy. Oral Microbiol Immunol.

[51] Balan P (2018). Keystone species in pregnancy gingivitis: a snapshot of oral microbiome during pregnancy and postpartum period. Front Microbiol.

[52] Ye C (2020). Unculturable and culturable periodontal-related bacteria are associated with periodontal inflammation during pregnancy and with preterm low birth weight delivery. Sci Rep.

[53] Yang I, Knight AK, Dunlop AL, Corwin EJ (2019). Characterizing the subgingival microbiome of pregnant African American women. J Obstet Gynecol Neonatal Nurs.

